# Computational processing of optical measurements of neuronal and synaptic activity in networks

**DOI:** 10.1016/j.jneumeth.2010.01.033

**Published:** 2010-04-30

**Authors:** Mario M. Dorostkar, Elena Dreosti, Benjamin Odermatt, Leon Lagnado

**Affiliations:** MRC Laboratory of Molecular Biology, Hills Road, Cambridge CB2 0QH, United Kingdom

**Keywords:** Calcium, Fluorescent reporter, Image analysis, Retina, SyGCaMP2, Software, Synapse, Zebrafish

## Abstract

Imaging of optical reporters of neural activity across large populations of neurones is a widely used approach for investigating the function of neural circuits in slices and *in vivo*. Major challenges in analysing such experiments include the automatic identification of neurones and synapses, extraction of dynamic signals, and assessing the temporal and spatial relationships between active units in relation to the gross structure of the circuit. We have developed an integrated set of software tools, named SARFIA, by which these aspects of dynamic imaging experiments can be analysed semi-automatically. Key features are image-based detection of structures of interest using the Laplace operator, determining the positions of units in a layered network, clustering algorithms to classify units with similar functional responses, and a database to store, exchange and analyse results across experiments. We demonstrate the use of these tools to analyse synaptic activity in the retina of live zebrafish by multi-photon imaging of SyGCaMP2, a genetically encoded synaptically localised calcium reporter. By simultaneously recording activity across tens of bipolar cell terminals distributed throughout the IPL we made a functional map of the ON and OFF signalling channels and found that these were only partially separated. The automated detection of signals across many neurones in the retina allowed the reliable detection of small populations of neurones generating “ectopic” signals in the “ON” and “OFF” sublaminae. This software should be generally applicable for the analysis of dynamic imaging experiments across hundreds of responding units.

## 1 Introduction

A major challenge in contemporary neuroscience is to understand the links between patterns of neural activity and behaviour or perception. To achieve this, it will be necessary to understand how neurones are connected within circuits, and then map the electrical and synaptic activity propagating through the circuit in relation to defined processing tasks. Electrophysiological approaches for monitoring activity across populations of neurones, such as multi-electrode recordings, offer a partial solution, but do not establish the identity and connectivity of recorded neurones, nor provide information about the patterns of synaptic activity that control spiking. An alternative approach is to assay neural activity using imaging techniques such as multi-photon microscopy, providing an opportunity to integrate studies of circuit structure and function.

The fluorescent molecules currently finding most widespread use as reporters of electrical activity detect changes in the cytosolic Ca^2+^ concentration (Garaschuk et al., 2006; Göbel and Helmchen, 2007; Rochefort et al., 2008). Such indicators include synthetic calcium dyes introduced by bulk-loading of tissue (Nimmerjahn et al., 2004; Kerr et al., 2005; Sullivan et al., 2005; Ohki et al., 2006; Mrsic-Flogel et al., 2007; Greenberg et al., 2008; Sumbre et al., 2008), and genetically encoded calcium indicators (GECIs) targeted to particular types of neurones by use of appropriate promoters (Tian et al., 2009; Wilms and Häusser, 2009). Calcium imaging allows electrical activity to be assayed in specific neuronal compartments, such as presynaptic terminals delivering the synaptic output (e.g. O’Donovan et al., 1993; Steele et al., 2005) and dendrites integrating synaptic inputs (Hausselt et al., 2007; Euler et al., 2008). Fluorescent reporters of synaptic vesicle exocytosis (Granseth et al., 2006; Miesenböck et al., 1998) and membrane potential (Sakai et al., 2001; Blunck et al., 2004; Chanda et al., 2005; Bannister et al., 2005; Perron et al., 2009; Gautam et al., 2009) also provide readouts of neural activity in intact circuits.

Now that it is possible to image dynamic signals across hundreds of neurones and synapses simultaneously, there is an important need to develop appropriate software for analysis (Ozden et al., 2008; Wilt et al., 2009; Mukamel et al., 2009; Grewe and Helmchen, 2009). Imaging experiments generate large volumes of data, and must be efficiently processed to define the large numbers of structures from which time-series signals can be measured. This multi-neuronal data should then be converted into forms allowing easy assessment by the scientist. The great advantage of imaging is that the positions and shapes of neuronal cell bodies, processes and synapses are recorded together with response dynamics, and this information should also be extracted to allow the investigation of function in relation to circuit structure. Simultaneous imaging of activity across hundreds of neurones also provides the opportunity to investigate how these act in concert, which should begin with the identification of units displaying correlated activity and discrimination between units that are functionally distinct. The format in which all this information is saved should facilitate meta-analysis across different experiments and data mining in the future, as well as allow for easy exchange of results between different laboratories. Unfortunately, these needs are not met by the general purpose image processing packages currently available.

**Fig. 1:**
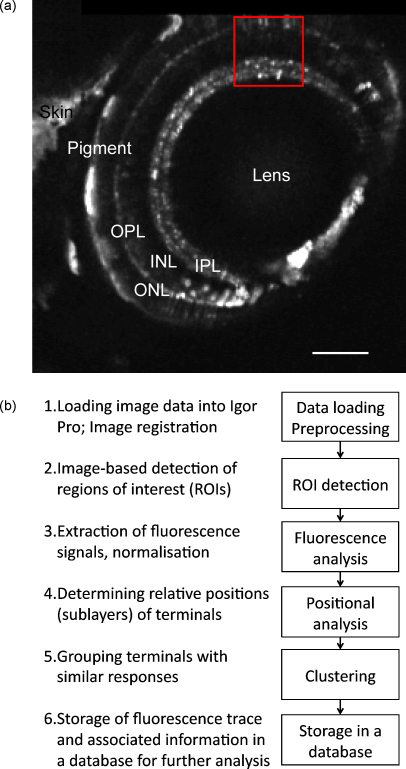
Outline of the analysis procedures. (a) The eye of a zebrafish larva 8 dpf expressing SyGCaMP2 in photoreceptor and bipolar cells was imaged on a multi-photon microscope at a resolution of 2.6×2.6×2μm/pixel. Data shown in Figs. 2, 3, 5 and 6 was recorded from the area delimited by the red box. INL, inner nuclear layer; IPL, inner plexiform layer; ONL, outer nuclear layer; OPL, outer plexiform layer; scale bar =100 μm. (b) Outline of the stages of analysis of optical recordings.

Here, we present a comprehensive collection of routines, which we named SARFIA (for Semi-Automated Routines for Functional Image Analysis) for the analysis of functional imaging data obtained using fluorescent reporters in intact neural circuits. These routines are brought together within a user-friendly environment provided by Igor Pro (Wavemetrics), a commercial data analysis package popular amongst electrophysiologists. We demonstrate the use of SARFIA to analyse the synaptic processing of visual information in the retina of zebrafish *in vivo* (Fig. 1a). The reporter we concentrate on is SyGCaMP2, a genetically encoded reporter of synaptic activity that detects the presynaptic calcium transient triggering neurotransmitter release (Dreosti et al., 2009). The main aims of this software are summarised in Fig. 1b and were (i) automated detection of structures from which dynamic signals are extracted, (ii) a quick and easy visualisation of activity across hundreds of labelled structures, (iii) determination of relative positions in a laminar circuit, (iv) implementation of clustering algorithms to identify structures with similar responses to a given stimulus, and (v) collecting the results in a database which can be screened for physiological and morphological properties (Fig. 1b). Because the software is designed to efficiently extract time-series information from fluorescence imaging data, it can also be used with conventional wide-field or confocal fluorescence microscopy and total internal reflection fluorescence (TIRF) microscopy.

## 2 Methods

### 2.1 Fluorescent reporter

All procedures involving animals were carried out according to the UK Animals (Scientific Procedures) Act 1986 and approved by the UK Home Office. We made transgenic zebrafish expressing the synaptically localised fluorescent calcium reporter SyGCaMP2 under a promoter specific for ribbon synapses (Dreosti et al., 2009, and Fig. 1a). Fish were kept at a 14:10 h light:dark cycle and bred naturally. Larvae were kept in 200 μM 1-phenyl-2-thiourea (Sigma) from 28 h post-fertilisation on to inhibit melanin formation (Karlsson et al., 2001).

For imaging, larvae 7–8 days post-fertilisation (dpf) were anaesthetised with 0.016% MS 222 (Sigma) and immobilised in 2.5% low melting agarose (Biogene) on a glass coverslip.

### 2.2 Imaging

**Fig. 2:**
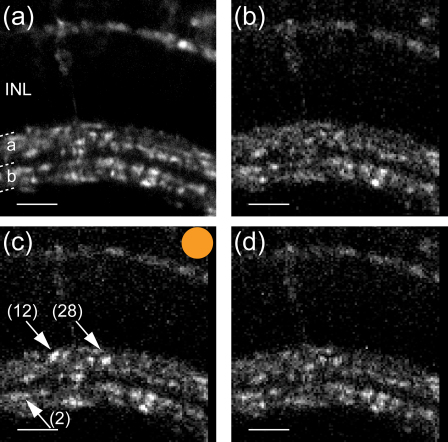
Synaptic terminals responding to a full field light stimulus. Selected frames of a recording from retinal bipolar cell terminals in the inner plexiform layer of the retina of a 8 dpf zebrafish expressing SyGCaMP2. (a) High resolution (512×512 pixels) overview of the area imaged. INL, inner nuclear layer; a, sublamina a of the inner plexiform layer; b, sublamina b of the inner plexiform layer. (b) A single frame from the original movie, recorded at 128×100 pixels at 5 Hz in the dark. (c) A single frame recorded while a full field amber light stimulus (13 mW/m^2^) was presented, indicated by an amber dot. Two terminals responding with an increase in fluorescence are marked by down-facing arrows, and two terminals in close proximity to each other responding with a decrease in fluorescence are marked with an up-facing arrow. The numbers next to the arrows correspond to the numbers of the respective ROIs in Figs. 4 and 6. (d) A single frame recorded 30 s after the light stimulus. Scale bars =25μm.

Retinae of transgenic fish were imaged *in vivo* using a custom-built 2-photon microscope (Tsai et al., 2002) equipped with a mode-locked Chameleon titanium–sapphire laser tuned to 915 nm (Coherent). The objective was an Olympus LUMPlanFI 40× water immersion (NA 0.8). Emitted fluorescence was captured by the objective and by a sub-stage oil condenser, and in both cases filtered by a HQ 535/50GFP emission filter (Chroma Technology) and a “hot mirror” that reflects wavelengths >700 nm (Edmund Optics) before detection by PMTs (Hamamatsu). Scanning and image acquisition were controlled using ScanImage v. 3.0 software (Pologruto et al., 2003) running on a PC. Light stimuli were delivered by amber and blue LEDs (Luxeon) projected through the objective onto the retina. Light stimulation was controlled through Igor Pro v. 4.01 software (WaveMetrics, Lake Oswego, OR) running on a Macintosh and time locked to image acquisition. Image sequences were acquired at 1 ms per line using 64×64 or 128×100 pixels per frame. Frames from a typical recording are shown in Fig. 2.

### 2.3 User interface

The software environment we have used to implement SARFIA is Igor Pro (version 6.1, Wavemetrics) which provides extensive signal and image processing capabilities, low-level programming control together with high-level analysis and presentational functions. The programming language of Igor Pro is learnt relatively easily. This environment is popular amongst electrophysiologists, with extensions such as Neuromatic and NClamp, Patcher’s Power Tools and Slice (all of which are freely available from http://www.wavemetrics.com/users/tools.htm). The custom control panels (Supplementary Fig. S1) allow point-and-click access to all major image manipulation, analysis and graphing functions.

### 2.4 Data import and pre-processing

Image stacks generated by ScanImage (.tiff format) were imported into Igor Pro and stored as single-precision floating-point arrays (which are termed “waves” in Igor Pro). ScanImage stores information such as the scanning speed, zoom factor, stage position, date and time of the experiment in the header of the .tiff files, and these were stored as “notes” associated with the respective waves. Since we had empirically measured the side length of an acquired image on our setup, the correct scaling of all three dimensions was calculated and immediately applied to the respective waves.

In many experiments, the images in a time-series were registered to correct for motion artefacts, which often occur during *in vivo* experiments (Greenberg and Kerr, 2009; Mukamel et al., 2009) using Igor Pro’s inbuilt image registration operation, which is based on an algorithm described by Thevenaz et al. (1998). Frames from a typical experiment representing raw data (after image registration) are shown in Fig. 2.

## 3 Analysis of multi-neuronal imaging experiments

### 3.1 Automated extraction of optical signals

The first stage of analysis in a dynamic imaging experiment is the definition of interesting structures within a field of view. Perhaps the simplest automated technique for fluorescence images is segmentation by thresholding, i.e. defining a region of interest (ROI) as a group of contiguous pixels whose brightness exceeds a criterion value (Burger and Burge, 2008, chap. 5.1). This approach is difficult to apply to intact neural tissue labelled with functional indicators: Differences in expression of reporter proteins or in uptake of synthetic dyes lead to differences in absolute brightness. If the threshold is set high enough to separate the bright cells or compartments in an image, dimmer regions are cut out. On the other hand, if the threshold is set low enough to detect dimmer regions, brighter units merge. For this reason, several recent studies imaging calcium signals within networks have resorted to the manual outlining of neurones recognised by eye (e.g. Niell and Smith, 2005; Göbel et al., 2007; Dombeck et al., 2007; Kerr and Denk, 2008), which is time-consuming and inconsistent. Recent attempts to automatically distinguish neurones from glia have focused on the faster calcium signals generated by neurones. For instance, Ozden et al. (2008) implemented a correlation-based method to identify pixels belonging to an active neurone which had been coarsely outlined by hand. Mukamel et al. (2009) further reduced the need for time-consuming user input by applying independent component analysis followed by segmentation to automatically assign signals to different cells. Both approaches calculated regions of interest from time-series data of changes in fluorescence, caused by neurones spiking independently from each other. Therefore, these methods defined active neurones very well, whereas non-spiking neurones were not picked up.

**Fig. 3:**
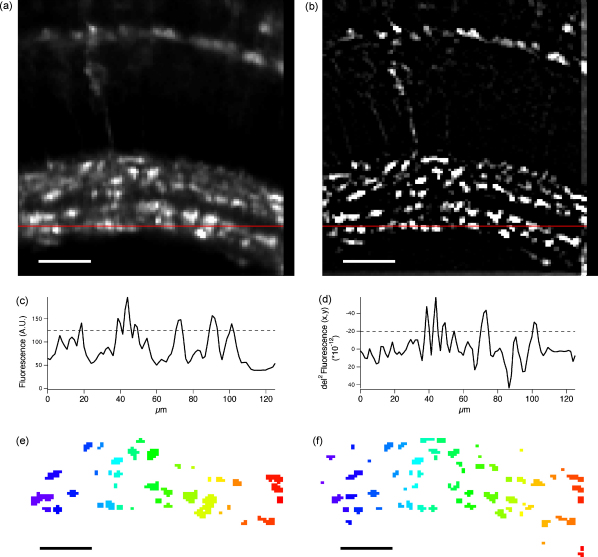
Thresholding using the Laplace operator. (a) Temporal average of the movie shown in Fig. 2. (b) Laplace operator of the image shown in (a). The image has been inverted (i.e. negative values are bright) and values ≥ 0 are black. (c and d) Line profiles measured from (a) and (b), respectively, as indicated by the red lines. The dashed lines represent thresholds required to detect the peaks between 40 and 55 μm. (e) ROI mask generated from the temporal average (a) using the threshold shown in (c). (f) ROI mask generated from the Laplace operator (c) using the threshold shown in (d). Scale bars =25μm.

One way to improve identification of neurones in calcium imaging experiments is to use GECIs targeted to cells of interest by use of appropriate promoters. Contrast in images is then enhanced by preventing the non-specific loading of glia. For instance, we targeted SyGCaMP2 to the ribbon-type synapses of photoreceptors and bipolar cells in the retina by use of the ribeye promoter (Dreosti et al., 2009). Nonetheless, neurones labelled with reporter proteins appear with varying degrees of brightness because of variations in expression levels and in their positions relative to the plane of focus (Figs. 2 and 3). To improve detection of units of lower contrast, we first transformed images using the Laplace operator, which is commonly used for edge sharpening in images (Burger and Burge, 2008, chap. 7.6). The Laplace operator of an Image I(x,y) is the sum of the unmixed second partial derivatives in the Cartesian co-ordinates *x* and *y* (Eq. (1)):

(1) $$\nabla^{2}I=\frac{\partial^{2}I}{\partial x^{2}}+\frac{\partial^{2}I}{\partial y^{2}}$$

Thus, areas that were brightest in the initial image had negative values in the transformed image (Fig. 3d and e). Segmentation was then carried out using a threshold calculated as a multiple of the standard deviation (SD) of all the pixel values in the Laplace operator. ROIs were then displayed to allow the user to make adjustments to the threshold with immediate feedback. In our experiments, we used values ranging from (−1.5) to (−4)× SD, and the value used in Fig. 3d was (−2.2)× SD.

Optionally, ROIs smaller than a chosen number of pixels could be rejected at this stage. This served as a lowpass filter for high resolution images and effectively reduced the number of traces that showed no signal.

The ROIs were numbered and stored in a matrix, termed “ROI mask” with the same *x* and *y* dimensions as the initial image sequence: Pixels that were outside ROIs were assigned a value of 1, whereas pixels inside an ROI were assigned a negative value based on their index number. A flowchart of the functions used to detect ROIs is given in Supplementary Fig. S2.

Detection of regions of interest was usually performed on the Laplace operator of a single image averaged over a certain period of recording in order to reduce noise and to detect as many labelled synaptic terminals as possible. Alternative starting points for the definition of ROIs currently provided by our software are detection of ROIs based on the standard deviation of the images in a time-series, or based on a “response image” displaying the change in intensity caused by a visual stimulus of known timing. The “response image” was calculated by subtracting the average of a user-defined number of frames recorded at rest from the average of a number of frames recorded during the stimulus. The precise number of frames averaged depended on the recording speed, the duration of the stimulus and whether short-lasting (transient) or long-lasting (sustained) stimuli were to be detected. The advantage of defining ROIs using the response image was improved detection of responsive terminals. The disadvantage was the immediate introduction of bias for a particular type of response and the rejection of neurones that were spontaneously active or which did not respond clearly to the particular stimulus we applied. In practise, we found that the most efficient and unbiased approach was to define ROIs corresponding to synaptic terminals using an average intensity image.

### 3.2 Quantification of optical signals

**Fig. 4:**
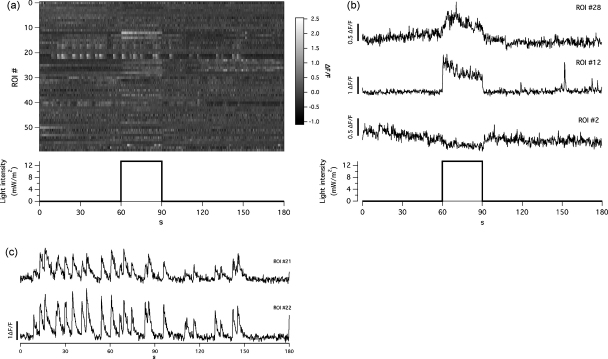
Spontaneous and light-evoked activity in the IPL. (a) Fluorescence data from 59 terminals, identified from the same set of data shown in Figs. 2 and 3. The lower graph shows the duration and intensity of the full field amber light stimulus. The numbers of the ROIs correspond to those shown in Fig. 6b. (b) Selected terminals responding to the light stimulus. ON responses (ROIs #12 and #28) show an increase in Ca^2+^ concentration during the light stimulus, OFF responses (ROI #2) a decrease. (c) Selected terminals showing spontaneous regenerative Ca^2+^ transients.

After defining structures of interest in a movie, the average intensity over each ROI was measured at each time-point and the results stored in a 2D wave indexed by the ROI number. Because ROI masks were saved independently, a definition of ROIs calculated from one movie could be used to analyse any other of the same width and height. A synopsis of this processing, that could be saved as a .pdf or .rtf file, was immediately produced. This contained a summary of the experiment, including imaging parameters, the averaged image in the field of view, the numbered ROI masks, raster plot of all signals on a grey-scale (Fig. 4a) and a series of graphs showing the time-dependent signal in each ROI (Fig. 4b and c).

Signals obtained using fluorescent reporters are often expressed as the relative change in intensity, ΔF/F, after correction for background signals arising from instrumentation and non-specific fluorescence (Yasuda et al., 2004). The background was determined by first manually specifying a “background” area in the image that was devoid of the reporter. Then, in each frame an average value for the background was calculated, which was subsequently subtracted from the respective frame (see also Supplementary Information).

Automatic determination of images defining a baseline ($F_{0}$) can be problematic if there is spontaneous activity, as we often observed in retinal bipolar cells of zebrafish larvae. Therefore, we defined the baseline as the bin centre of the highest peak of a histogram of all fluorescence values in a given trace. The number of bins was $1+\log_{2}$ *N*, where *N* was the number of points in a given trace. This approach performed well for the levels of spontaneous activity that we encountered but will overestimate the value of $F_{0}$ if there are high levels of activity.

### 3.3 Sorting functional classes of response by hierarchical clustering

**Fig. 5:**
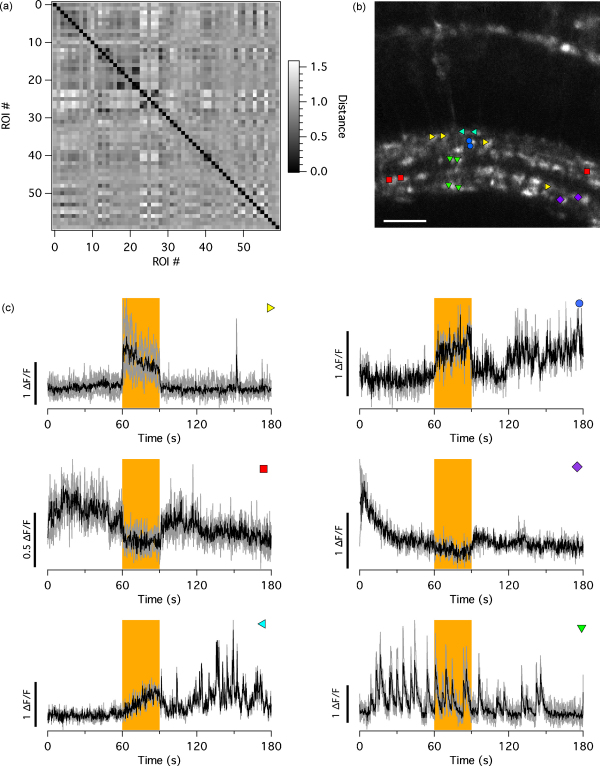
Hierarchical clustering to sort traces. (a) Distance matrix showing correlation between the traces shown in Fig. 4. The distance was calculated as 1− Pearson’s *r*. (b) The positions of terminals in six distinctive clusters are overlaid on an overview of the recorded region. Scale bar =25μm. (c) Traces (grey) and averages (black) of six distinctive clusters. Amber bars indicated when a full-field light stimulus was presented. The symbols in the upper right corner correspond to the symbols in (b).

Retinal bipolar cells respond to a given light stimulus in a stereotypical way, e.g. combinations of ON/OFF and transient/sustained in response to a full field light stimulus (Figs. 4 and 5). Initially, we grouped these manually, but ideally we wanted to avoid forming these groups by eye, and rather use an objective approach. Therefore, we implemented hierarchical clustering algorithms, which are usually employed in the analysis of microarray data (Gollub and Sherlock, 2006). Clustering methods have been used before to identify neurones firing in synchrony. Ozden et al. (2008), for instance, successfully used repeated runs of a *k*-means algorithm (termed meta-*k*-means) to cluster groups of cerebellar dendrites that fired together, while Eggermont (2006) used hierarchical clustering to discern clustered activity of neurones in the auditory cortex in response to acoustic stimuli. We have used a similar approach, implementing a form of hierarchical clustering to distinguish groups of calcium traces.

Traces were compared pair-wise to yield a distance matrix (Fig. 5a) using one of the following metrics: Pearson’s *r*, Euclidean, normalised Euclidean, Manhattan, or Chebychev (see Gollub and Sherlock, 2006, for a detailed description of these metrics). The Pearson distance was calculated as (1− Pearson’s *r*), so that, in analogy to the other metrics, a distance of 0 indicated perfect correlation. Then, a cutoff was chosen as a percentage of the highest distance (typically 25–30%). Any two traces that had a distance to each other of less than the cutoff were grouped into an initial cluster. Further traces were added by single linkage (or nearest neighbour clustering) to this cluster, i.e. if their distance to any of the traces in this cluster was less than the cutoff. We generated clusters from the data shown in Fig. 5 using the Pearson distance and a cutoff of 25%. This resulted in six distinct clusters with two to four members, highlighting groups ON and OFF terminals with different kinetics, as well as a group of simultaneously spiking terminals (Fig. 5b and c). Noticeably, two different clusters of ON, OFF and spontaneously active terminals, respectively, with different kinetics or spiking patterns were detected.

## 4 Extracting positional information

Many areas of the nervous system are organised into distinct layers, including the cerebral cortex, hippocampus, cerebellum and retina. In the 19th century, Ramón y Cajal attributed this to the “law of space conservation”, which states that somata and processes of cells would occupy the least possible amount of space (Ramón y Cajal, 1995). Thus, the function of neural circuits is intimately related to their structure (e.g. Bell et al., 2008; Canto et al., 2008; Silberberg, 2008).

Often, a neurone will receive synaptic inputs in one layer, but deliver its output in another. In the inner retina, visual signals are transferred from bipolar cells to ganglion cells in the inner plexiform layer (IPL), which is composed of 5–6 substrata (Connaughton and Nelson, 2000; Connaughton et al., 2004; Mumm et al., 2006; Nevin et al., 2008). It is thought that different functional classes of ganglion cell collect different signals in these strata before sending the results of retinal processing to the brain (Masland, 2001). We thus sought to define the position of all the bipolar cell terminals we imaged with reference to the layered structure of the IPL. A simple metric based on the radial depth of a terminal through the IPL, with 0% at the margin closest to the ganglion cell layer and 100% at the margin closest to the photoreceptors has been used before (e.g. Connaughton et al., 2004; Mumm et al., 2006). We therefore developed a semi-automatic approach for defining the positions of all terminals in this way.

**Fig. 6:**
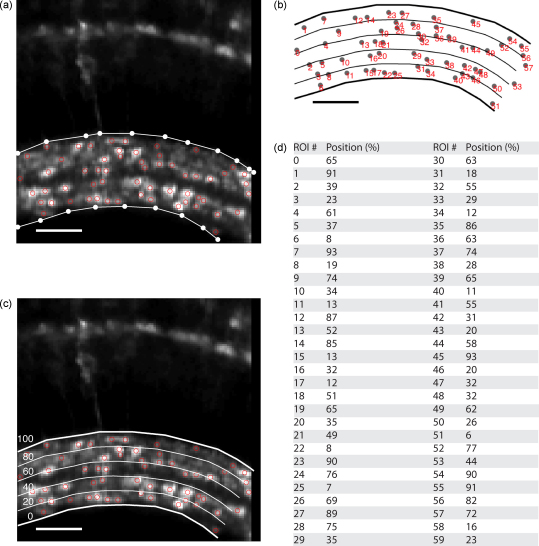
Determinig positions within the IPL. (a) Centres of mass of the ROIs determined as shown in Fig. 3 (red circles) and manually outlined borders (white dots). (b) Index numbers of the ROIs, corresponding to the traces shown in Fig. 4a. (c) Interpolated and smoothed borders (bold white lines) and four representative isocontours (thin white lines). (d) Table of relative positions. Scale bars =25μm.

Terminal positions were calculated based on the ROI mask and the average image (or the standard deviation image or the response image, depending on which one was used to determine the ROIs). First, the inner and outer limits of the IPL were outlined. Automatic detection of the limits by thresholding worked reliably only if no other bright structures (such as the outer plexiform layer and a cell body in Fig. 6a) were present in the picture. Therefore, we outlined the contours manually in most experiments (Fig. 6a), which yielded comparable results. These contours were then interpolated to have the same number of points (typically 8× the number of pixels per line) and smoothed using a locally weighted polynomial regression algorithm (Cleveland, 1979). The contour facing the photoreceptors was defined as 100% ($C_{100}$) and the contour facing the ganglion cells was defined as 0% ($C_{0}$). Between them 99 isocontours ($C_{i}$) were calculated according to Eq. (2) (Fig. 6b):

(2) $$C_{i\left(0\ldots 100\right)}=\frac{i\times C_{100}+\left(100-i\right)\times C_{0}}{100}$$

Two more isocontours, −1% and 101%, were extrapolated so that terminals outside the limits were binned into the respective group. Then, centres of mass ($\vec{R}$) were calculated for all ROIs by weighing the vector of each pixel of the ROI ($\vec{r_{i}}$) by its respective brightness (or “mass”, $m_{i}$; see Eq. (3) and Fig. 6c):

(3) $$\vec{R}=\frac{\sum \left(\vec{r_{i}}\times m_{i}\right)}{\sum m_{i}}$$

Finally, Euclidean distances between the isocontours and each centre of mass were calculated. The index i of the isocontour that had the shortest distance to a centre of mass was assigned that centre of mass’ relative position (Fig. 6d). A flowchart of the functions used to determine positions is given in Supplementary Fig. S3.

**Fig. 7:**
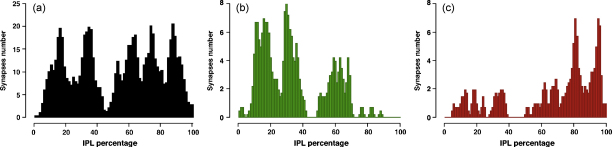
Distribution of synaptic terminals in the IPL. (a) Distribution of more than 400 bipolar cell terminals in the IPL, recorded *in vivo* from zebrafish larvae (8 dpf) expressing SyGCaMP2. (b) Subset of terminals that responded with a depolarising (ON) response to a light stimulus. (c) Subset of terminals that responded with a hyperpolarising (OFF) response to a light stimulus.

When we plotted the positions of > 400 terminals of 8 dpf zebrafish larvae, we could distinguish at least 5 distinct sublayers, 3 in sublamina a and 2 in sublamina b (Fig. 7a), which corresponds well with previous publications (Connaughton and Nelson, 2000; Connaughton et al., 2004; Mumm et al., 2006; Nevin et al., 2008). By assessing changes in calcium concentrations at synaptic terminals during and immediately after applying a spatially uniform light stimulus, we classified responding terminals into ON (increase during the light stimulus) or OFF (decrease during and/or increase immediately after the stimulus) and plotted histograms of the positions of the respective terminals (Fig. 7b and c). ON terminals were distributed in sublamina b and the innermost layers of sublamina a, whereas the majority of OFF terminals were found in the outer two layers of sublamina a. However, we also found a smaller subpopulation of “ectopic” OFF terminals in sublamina b that had not been detected previously. Together with recent publications (Dumitrescu et al., 2009; Hoshi et al., 2009), these findings challenge the notion that “the axons of OFF and ON cone bipolar cells terminate at different levels (strata) within the IPL: OFF in the outer half, ON in the inner half” (Wässle, 2004).

## 5 Discussion

In this report we describe functions we used to analyse data from *in vivo* calcium imaging in the retina of zebrafish larvae using the novel, synaptically localised fluorescent calcium indicator SyGCaMP2 (Dreosti et al., 2009). We improved the detection of ROIs of varying brightness over conventional methods and implemented algorithms to determine the positions of ROIs relative to the width of the IPL. Furthermore, we implemented hierarchical clustering algorithms to organise the resulting data and built a database to combine the analyses of a large number of separate experiments.

### 5.1 Implementation in Igor Pro

We wrote the functions described in this report in Igor Pro, a commercial data acquisition and analysis program, in which custom functions can easily be programmed. However, the approaches described here do not depend on a specific software and can be implemented in a range of commercial and open-source computing environments. The same holds true for the software used to acquire the images. We preferred Igor Pro for analysis because it allows access to inbuilt and custom-written functions via a graphical user interface in addition to a command-line (Supplementary Fig. S1), which facilitated accessibility by users less familiar with the program.

### 5.2 Advantages and disadvantages of using the Laplace operator for detection of ROIs

We implemented ROI detection based on the Laplace operator because of the strong variation in brightness of synaptic terminals in our specimens (see Figs. 2 and 3a and b). As a second derivative, the Laplace operator highlights changes in relative brightness rather than absolute brightness (Fig. 3c and d) and therefore facilitates the detection of regions that are brighter than their immediate background, rather than a background value based on the entire image. Similar results have been achieved previously by calculating the à trous wavelet transform decomposition of an image (Olivo-Marin, 2002). While the results using either method on our data were similar, we found that using the Laplace operator was somewhat easier to apply, since the user had to adjust only a single value (the threshold). In the case of the à trous wavelet transform decomposition, in contrast, we needed to adjust the deepest level of correlation, the threshold to filter the wavelet coefficients and the detection level (*J*, *k* and $l_{d}$, respectively, in Olivo-Marin, 2002) in order to obtain optimal results.

ROI detection based on the Laplace operator works well as long as the cross-section of ROIs have peaks (Fig. 3d). However, if the cross-section flattens out, only the edges will be picked up, but not the centre of the ROI, which may become apparent with images taken at high resolution (i.e. <1 μm^2^/pixel in sections of the IPL). Practically, we overcame this by first filtering the high resolution image using a Gaussian filter and then creating a lower resolution (pixelated) image from which ROIs were detected. The so resulting ROI mask was then resampled (without interpolation in order not to change the values of the ROIs) back to the resolution of the original image. However, ROIs generated this way had much coarser features than the original high resolution image. Alternatively, image closing (binary erosion, followed by dilation; Burger and Burge, 2008, chap. 10.3) could be implemented, although this operation risks merging of closely spaced ROIs. Thus, thresholding the Laplace operator is particularly well suited for the detection of ROIs of variable brightness as long as the cross-section of an ROI has a peak.

The main advantage of this method over using a correlation-based method (Ozden et al., 2008) was that we could limit user input to adjusting a single value (the threshold), rather than having to outline each of tens to hundreds of structures manually. Independent component analysis (ICA; Mukamel et al., 2009), in contrast, performed better on sparsely spiking neurones than on slow glial calcium waves. However, slow calcium signals that can last for tens of seconds (Fig. 4) are typical for retinal bipolar cells. Furthermore, ICA was able to separate signals from sources that overlapped in the imaged area. Since we used a genetically targeted sensor, we encountered far less mixing of signal sources, which we reduced even further by minimising the thickness of the excitation volume. Therefore, we were able to extract high quality signals from our data using an image-based segmentation approach.

### 5.3 Correlating structure with function

Different types of information, such as opposing polarities (ON/OFF) or temporal properties (transient/sustained) are transmitted in “parallel pathways” in the vertebrate retina (Masland, 2001). In the retina of zebrash, 17 different types of bipolar cells were identified based on morphological (Connaughton et al., 2004) and electrophysiological (Connaughton and Nelson, 2000) properties. In the inner plexiform layer, bipolar cells make synaptic contacts to amacrine and ganglion cells in 5–6 distinct sublayers, whose position is usually expressed as a percentage of the width of the IPL (Connaughton et al., 2004; Mumm et al., 2006). We determined positions in the IPL by calculating isocontours from the outlines of the IPL and matching them to the centres of mass of the ROIs. Then we separated the terminals depending on the polarities of their responses and plotted these results in histograms (Fig. 7).

However, this approach was feasible only if the optical section cut orthogonally through the layered structure. This was relatively easy to achieve in the retina, as it roughly describes a hemisphere. Thus, to calculate statistics on the position of terminals, we used only data from focal planes where a wide gap between sublaminae a and b was visible, which indicated good alignment of the inner plexiform layer with the imaging system. For preparations of other tissues, however, careful positioning of the tissue or experimental animal may be warranted. In contrast, detecting ROIs and extracting time-series information from images whose focal planes were tilted respective to the neural layering was not impaired.

Similarly, we plan to map not only the polarities of responses, but also responses to different temporal qualities, colours or contrast in order to obtain a more complete picture of the distribution of these functional aspects in the inner plexiform layer.

### 5.4 Clustering

Clustering methods have been used before to identify neurones firing in synchrony. Ozden et al. (2008), for instance, successfully used repeated runs of a *k*-means algorithm (termed meta-*k*-means) to cluster groups of cerebellar dendrites that fired together. We have used a similar approach, implementing a form of hierarchical clustering to group the stereotyped responses of retinal bipolar cells. The same functions, however, may also be used to cluster the signals recorded from separate pixels in the original image stack, in order to decide whether they should be included in a particular ROI (as in Ozden et al., 2008, for instance).

While this approach was computationally straightforward to implement, the cutoff to group traces into clusters was set somewhat arbitrarily. The disadvantage of the *k*-means approach, in contrast, is the uncertainty in choosing an optimal number and arrangement of partitions (Gollub and Sherlock, 2006). Ozden et al. (2008) overcame this by using the computationally more demanding approach of calculating meta-*k*-means.

Applying a hierarchical clustering algorithm to our data yielded six clusters of closely correlated traces (Fig. 5c). Combined with the positional information (Fig. 5b), we wondered whether traces in the same cluster originated from the same neurone. Given that bipolar cells have been reported to spike spontaneously, even when isolated from the retina, (Burrone and Lagnado, 1997; Ma and Pan, 2003) and thus most likely spike independently from each other, the answer would be “probably yes” for the cluster of spontaneously spiking terminals (green triangles in Fig. 5b and c). However, terminals grouped into a cluster of OFF responses (red squares in Fig. 5b and c) were too distant and did not match any previously described branching pattern (Connaughton et al., 2004) and thus were unlikely to belong to the same neurone. Therefore, we were not able to sufficiently address this question using the results from the clustering algorithm alone. Combined with a sparse labelling approach, however, such as transient expression of a fluorescent protein in a genetically targeted subset of bipolar cells in combination with functional imaging, we should be able to identify the terminals of a particular bipolar cell with greater fidelity.

### 5.5 Summary

Our ongoing experiments directly drive the implementation of new computational methods to improve or facilitate their analysis. For instance, the data to generate Fig. 7 had originally been classed by eye and copied manually into tables from which we made the histograms. This prompted the notion of building a database from which such information could be extracted automatically (which is described in the Supplementary Information). Thus, while compiling our first histograms illustrating the functional anatomy of the inner retina had been a major effort, we are now able to generate such figures from vast datasets, or subsets of those, within minutes.

Lastly, we wanted to make not only the computer code itself, but also the ideas behind them and our practical experiences public, so that the same steps won’t have to be retaken wherever similar experiments are being conducted.

## Appendix B Availability of the software

SARFIA is available free of charge and can be downloaded from the Igor exchange, http://www.igorexchange.com/project/SARFIA. A fully functional demo version of Igor Pro is available from the company's website, http://wavemetrics.com/. The core functions of SARFIA, thresholding and extraction of time-series information, are also available as Matlab code from the Matlab file exchange, http://www.mathworks.com/matlabcentral/fileexchange/, file ID #26482.
