# Supplementary material for: Computational processing of optical measurements of neuronal and synaptic activity in networks
Source: J Neurosci Methods. 2010 Apr 30;188(1):141–50. doi: 10.1016/j.jneumeth.2010.01.033 (PMC2849931; doi:10.1016/j.jneumeth.2010.01.033)
Supplement: Supplementary file 1 [file mmc1.pdf]

Supplementary information
(Computational Processing of Optical Measurements of Neuronal and Synaptic Activity in Networks)

Mario M. Dorostkar, Elena Dreosti, Benjamin Odermatt, Leon Lagnado

MRC Laboratory of Molecular Biology
Hills Road, Cambridge
CB2 0QH, United Kingdom

1. Normalisation of fluorescence data

In order to compensate for different expression levels of the reporter protein or different loading of dyes, we attempted to normalise fluorescence data to a baseline ($\Delta F/F$; Eqn. 1), where $F(t)$ is the recorded fluorescence at time t, F_0 is the fluorescence at baseline and $F_{BG}(t)$ is the background fluorescence at time t.

$$\Delta F/F = \frac{F(t) - F_0}{F_0 - F_{BG}(t)} \tag{1}$$

However, this approach had two possible pitfalls – determining the baseline (F_0) and the background fluorescence ($F_{BG}(t)$): Determining a baseline is often straightforward, usually the baseline is defined as the fluorescence before (or after) a stimulus. However, in *in vivo* recordings in retinal bipolar cells, we regularly encountered spontaneously active terminals (Fig. 6c in the main text) that did not respond to a stimulus. Yet, we wanted to use an automated approach that worked in all kinds of traces that we recorded.

Therefore, we defined the baseline as the bin centre of the highest peak of a histogram of all fluorescence values in a given trace. The number of bins was $1 + \log_2 N$, where N was the number of points in a given trace. This approach performed well for the levels of spontaneous activity that we encountered as well as for the stimuli that we used. However, this approach may falsely detect a baseline with long stimulus durations or high levels of spontaneous activity.

The fluorescence background is composed of shot noise from the photodetectors, the intrinsic noise of the recording equipment and photons reaching the photodetectors that are not emitted fluorescence. The latter was particularly pronounced when we gave light stimuli, even though the wavelengths used (455 and 590 nm) were supposed to be blocked by the emission filters (HQ535 $\Delta F50$, Chroma Technologies). We subtracted the background by manually specifying an area in the image that was devoid of the reporter. In each frame, the average fluorescence from that area was calculated and subtracted from the whole frame. Alternatively, Igor Pro can calculate a polynomial fit of the background, which can be subtracted from frames instead of a scalar value, in order to compensate for non-uniform background values. Practically, we encountered weak fluorescence in the whole visual field (which can be visualised by adjusting the contrast settings of the displayed graph), which led us to slightly overestimate the background signal and thus also the $\Delta F/F_0$ signals. Therefore, one has to exercise due care when comparing amplitudes of signals from different recordings (Yasuda et al., 2004).

2. Building the database

A single experiment was able to generate data from tens, even hundreds, of synaptic terminals. It was soon obvious that we wanted to perform meta-analyses of different experiments and even exchange data between experimenters as each experiment contained a wealth of data that could be analysed to answer different questions. Therefore, we constructed a special format to store fluorescence data as well as associated information of the trace, such as position and size of the terminal, age of the test subject, categories (e.g. ON/OFF, transient/sustained), the baseline and background fluorescence, or the parameters for the analysis.

The data was stored in a two-dimensional array, each column holding the information of a particular trace. Each column would first hold the raw, or background-subtracted fluorescence data, followed by a footer that held numeric information such as the number of the ROI in the original context, size of the ROI (in m^2), the number of points of the original recording and the scale factor, or the fluorescence level of the baseline (Tab. 1). Non-numerical data such as the units (usually seconds) or the name of the original file were converted to numbers either by storing the index of the data from a particular list. For instance a list held the abbreviations for all SI units (e.g. "s;g;m;mol;A;..."). The index for seconds, the first item in the list, was 0, hence the entry in the field "XUnits" in the database. Strings were converted to numbers in a similar fashion: Each character was converted to a number by looking up its index in a list multiplied by the number of characters in the list to the power of the position of the character in the string, as lined out in Eqation 2, where *length* is is the number of characters in the string, *base* is

the number of characters in the list and *index* is the index of the character in the list.

$$StringtoNumber = \sum_{i=0}^{length-1} base^{length-1-i} \times index \qquad (2)$$

As this method rapidly generates very large numbers (in the order of $\sim 10^{15}$ for strings with 10 characters), it is suitable to store and recreate only strings of up to 10 characters, which was, however, sufficient for our use.

Storage and retrieval of data made use of Igor Pro's feature of "dimension labels", which allow to access a point in a wave (an array) by its label, regardless of the absolute position in the wave. Thereby, we were even able to add fluorescence traces with different numbers of points in the database.

Table 1: Layout of the database

**Label**	**ROI** 1	**ROI 2**	...	**ROI** n
Time 0	105.803	148.803		65.8034
Time 1	178.678	118.181		25.2758
⋮				
Time n	50.2094	97.8788		126.089
ROINr	0	1		59
Age	8	8		8
Position	62	89		24
Size	1.4e-12	5.5e-12		2.8e-12
ONOFF				
TSus				
Stim				
BaseLine	57.0765	86.1964		76.4
AnalysisBitMask	12.2	12.2		12.2
nPoints	900	900		900
XDelta	0.2	0.2		0.2
XOffSet	0	0		0
XUnit	0	0		0
OriginID	2.15e+15	2.15e+15		2.15e+15

4. Supplementary figures

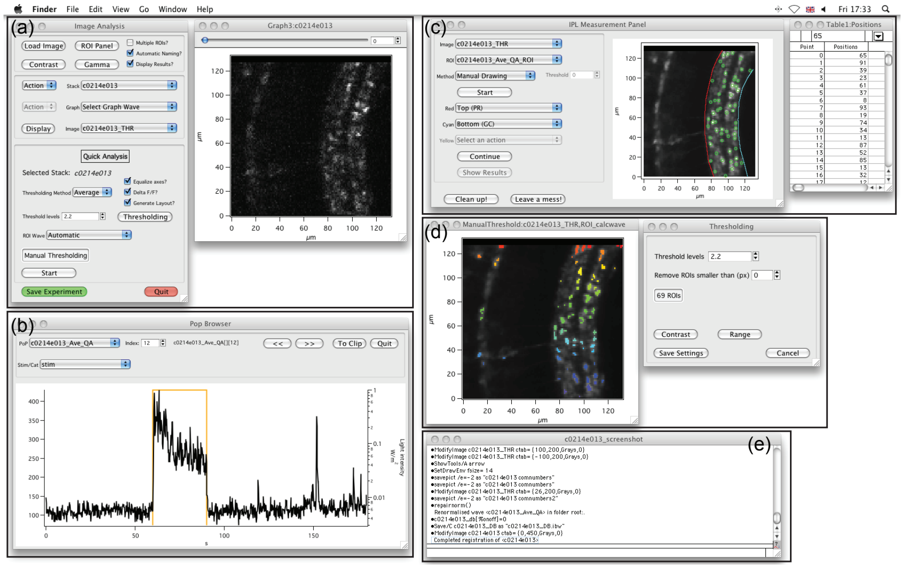

Figure 1: **Screenshot of the SARFIA custom control panels.** (a) The main panel to access and analyse images (left) and a preview of the loaded image stack (right). (b) Browser to display single traces, overlaid with the stimulus pattern (amber). (c) Control panel to determine positions (left) and results displayed in a table (right). (d) Control panel for determining regions of interest (right) and preview of the current settings (left). (e) Command window.

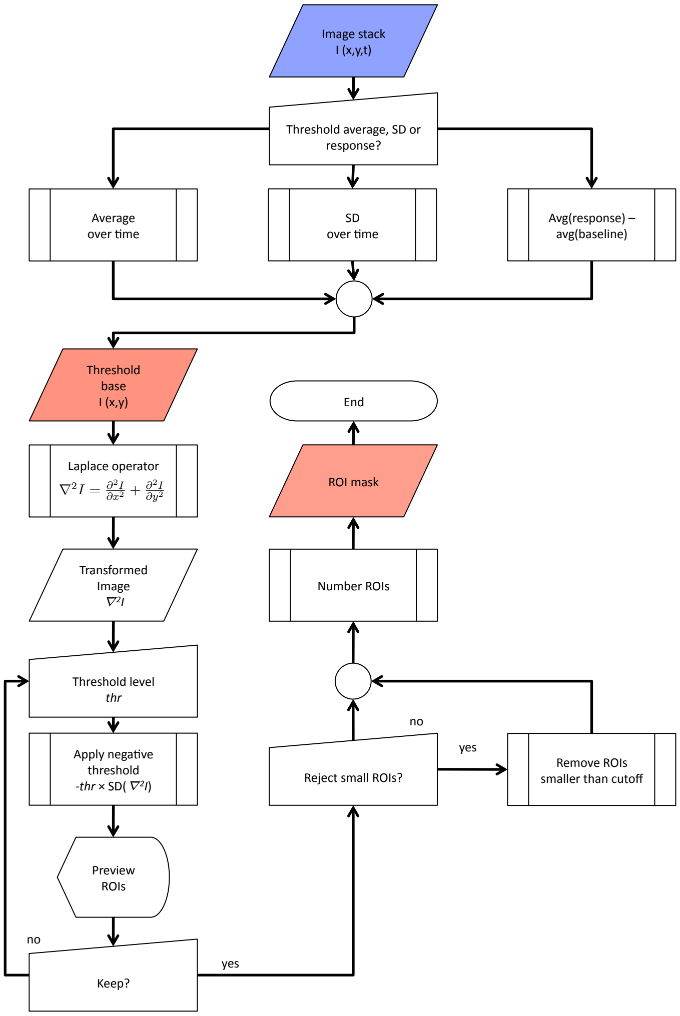

Figure 2: **Flowchart of the thresholding functions.** Blue polygons represent inputs, and red polygons represent the outcomes. SD, standard deviation; avg, average;

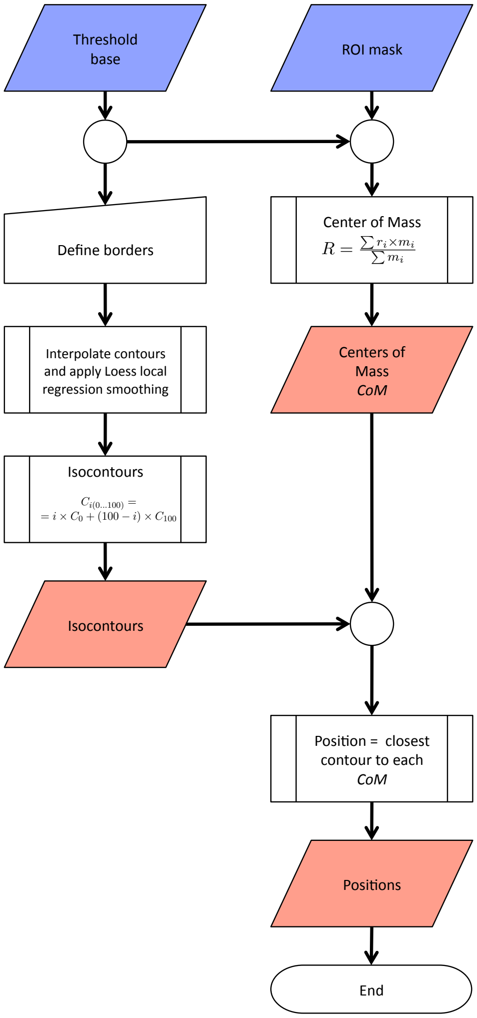

Figure 3: **Flowchart of the positioning functions.** Blue polygons represent inputs, and red polygons represent the outcomes.
